# A framework for evaluating e-health: Systematic review of studies assessing the quality of health information and services for patients on the Internet

**DOI:** 10.2196/jmir.2.suppl2.e13

**Published:** 2000-09-13

**Authors:** Gunther Eysenbach

## Abstract

**Context:**

A recent concern and topic of many publications in the last three years has been the quality of health information and services for the public on the Internet.

**Objectives:**

To identify and summarize studies published in the peer-reviewed literature evaluating the quality of information and services for consumers on the Internet, including information published on web sites, information on newsgroups and mailing lists and other venues such as email contacts with doctors, as well as studies evaluating the quality of ehealth services such as cyberdoctors and cyberpharmacies.

**Data Sources:**

MEDLINE and PREMEDLINE (1966 - May 2000), Science Citation Index (1992-May 2000), Social Sciences Citation Index (1992- May 2000), Arts and Humanities Citation Index (1992-May 2000) and a personal bibliographic database.

**Study Selection:**

We included empirical studies where investigators searched the Internet systematically for specific health information or clearly define a set of specific services to be included, evaluated the quality of information or services found, and reported quantitative data.

**Data Extraction:**

Study characteristics, medical domain, search strategies used, quality criteria and methodology of quality assessment, results (number of sites rated as sufficient pertaining to a quality), quality and rigor of study methodology and reporting.

**Data Synthesis:**

A total of 41 studies met the inclusion criteria, dealing either with content of websites, information on e-commerce sites, quality of online-care or community venues. A) Content: 29 evaluated information on websites, of those 5 evaluated information on websites from the field of pediatrics, 3 from oncology, 3 pharmacology information, 2 nutrition information, 4 general clinical information and 12 specific information from other clinical disciplines. Studies varied widely in methodology, quality and results. Among the 29 studies dealing with quality of health information on websites, one study evaluated the authority of source, 19 studies checked sites for presence or absences of technical criteria (such as disclosure of sponsorship, authorship, presence of references or last update), three evaluated readability, 20 evaluated the accuracy of information, and 12 content completeness. B) E-Commerce: Three studies dealt with drug information on e-commerce sites. To evaluate the quality authors extracted prices, checked for completeness of online-history taking and/or information provided on the site e.g. pertaining to contraindications or the presence/absence of disclaimers and/or liability waivers. Only one of these three studies actually used the service by ordering drugs, allowing to evaluate quality of online-advice, qualification of cyberdocs, delivery time, reasons for non-delivery. C) Care: Five studies dealt with the quality of online consultations, of them one dealt with advice given by ordinary physicians in response to a unsolicited fictionous patient request, two evaluated the responses of cyberdocs soliciting patient requests, and two evaluated advice given in response to a request for an online prescription directed to drug e-commerce sites. D) Community: Seven studies evaluated messages on mailing lists or usenet newsgroups. Two studies collected messages from a venue and evaluated them for accuracy, two studies posted a "test" message on a newsgroup and evaluated the accuracy of responses. In three studies authors used the cumulative impact factor of the published research of the mailing list contributors as an indicator for the qualification of the authors and thus as quality indicator of the mailing list.

Of the 23 studies evaluating accuracy and/or completeness of information provided on websites, 10 used guidelines as gold standard (all of the to extract a-priori criteria), five used peer-reviewed literature (all to compare information a posteriori), two used textbooks, one used consensus among the raters and three used the personal experience or opinion of the author. All three studies which compared information against personal opinion came to a positive conclusion regarding the accuracy of information, while all of the more rigorous studies comparing information against guidelines concluded that much of the information found on the web were of low quality. In two cases it was not reported where the gold standard comes from. 9 studies used more than one rater to assess a website, 6 of them provided some sort of information on inter-observer variability.

**Conclusions:**

Methodology, results and conclusions of investigators vary widely. There is a wide variability even regarding the evaluation of formal criteria such as authorship and references. Differences in study conclusions regarding the quality of Internet information are likely a result of difference in study rigor, evaluation criteria and topic chosen. All but five studies concluded that quality is a problem on the Internet. The rigor of these five studies coming to a more positive conclusion as expressed in a "assessment quality score" was significantly inferior to the remaining studies.

Although there were two comparative studies of the quality of information on the Internet compared with information found outside of the Internet, there is little evidence that health information found on the Internet is worse than health information in traditional media.

A conceptual and methodological framework is presented for describing, comparing, and analyzing the structure and quality of e-health, based on Donabedians quality measures of structure, process and outcome.

*(full paper submitted to a peer-reviewed journal)*

**Table 1:** Proposed conceptual and methodological framework for describing, comparing, and analyzing the structure and quality of e-health

	*Structural Quality*		*Process (Performance) Quality*	*Outcome Quality*
What do we want to evaluate / improve?	Communication setting, infrastructure, resources		Communication process itself	Effect of communication
	*Real structure*	*Virtual Structure*		
Evaluation Level	Level 1	Level 2	Level 3	Level 4
Unit of evaluation	Information providers	Websites and webpages, or other Internet venues	Medical advice and support given, messages and statements made	Users
What can external evaluators assess?	Technical capabilities of ehealth providers, way of presentation, completeness of disclosure/metainformation provided		Quality of advice and standard of ehealth care (evaluating information)	Impact on patients
Aims of measures directed to improve quality	Providing access and facilitating communication Helping users to find and to navigate Building trust Making the information context clear for the user (disclosure etc.) Enabling informed consent Providing efficient feedback channels Giving insight into the editorial process and enable checking		Acting in line with clinical and ethical guidelines	Improving patient outcome
Methods of evaluation	Obtaining information from the information provider	Checking for presence of technical criteria	Checking the information content for accuracy, Testing the service and comparing advice against guidelines	Obtaining outcome variables from patients
Criteria	Level 1	Level 2	Level 3	Level 4
	Resources (capital, infrastructure)	Ease of access	Actual accuracy (includes currency and completeness) of content	Mortality
	Staff (number, qualification, leadership)	Speed	Accuracy of advice	Morbidity
	Training	(Readability)	Ethical behavior, including privacy, confidentiality	Quality of life
	Internal Standard Operating Procedures and quality assurance processes, commitment to quality	Disclosure	Validity of health risk assessment tools	Cost effectiveness
		Attribution		(Behaviour change, change in attitude and knowldege)
		Displaying the date		
		Clarifying the target population		
		Accountability		
		Indirect measures: Popularity, number of links pointing to the site		
Information about compliance with criteria accessible for	Information provider	User, particularly consumers	Experts	Researchers in collaboration with information provider
Universality of quality criteria	Universal criteria	"General" quality criteria, specific to the Internet venue and (partly) aim	"Subject specific" quality criteria, specific to the medical domain	Universal criteria, but quantifiable outcome measures specific to the aim

